# Nationwide cross-sectional study of antimicrobial stewardship and antifungal stewardship programs in inpatient settings in Japan

**DOI:** 10.1186/s12879-021-06035-5

**Published:** 2021-04-16

**Authors:** Yuki Moriyama, Masahiro Ishikane, Yoshiki Kusama, Nobuaki Matsunaga, Taichi Tajima, Kayoko Hayakawa, Norio Ohmagari

**Affiliations:** 1 Disease Control and Prevention Center, National Center for Global Health and Medicine, 1-21-1 Toyama, Shinjuku-ku, Tokyo, 162-8655 Japan; 2 Collaborative Chairs Emerging and Reemerging Infectious Diseases National Center for Global Health and Medicine Graduate School of Medicine , Tohoku University , 1-1, Seiryo-machi, Sendai-shi, 980-8574 Aobaku, Miyagi, Japan; 3 AMR Clinical Reference Center, National Center for Global Health and Medicine, 1-21-1 Toyama, Shinjuku-ku, Tokyo, 162-8655 Japan

**Keywords:** Antimicrobial stewardship, Antifungal stewardship, Inpatients, Japan

## Abstract

**Background:**

To prevent antimicrobial resistance, both antimicrobial stewardship (AMS) and antifungal stewardship (AFS) in inpatient settings are needed in small/middle-sized hospitals as well as large hospitals.

**Methods:**

We conducted the web-based, self-administered, nationwide cross-sectional study regarding AMS and AFS in inpatient settings in Japan, targeting hospitals that participated in a hospital epidemiology workshop conducted in July 2018. The questionnaire was composed of intervention protocols for use of broad-spectrum antimicrobials and antifungals within 7 or 28 d of beginning usage. These broad-spectrum antimicrobial and antifungal protocols were compared between large (≥501beds) and small/middle-sized (≤500 beds) hospitals.

**Results:**

Of 240 hospitals surveyed, 39 (16%; 18 large and 21 small/middle-sized) responded. The number of hospitals that intervened in the use of broad-spectrum antimicrobials within 7 and 28 d were 17 (44%) and 34 (87%), respectively; those that intervened for antifungals were 3 (8%) and 10 (26%), respectively. Interventions for use of broad-spectrum antimicrobials within 7 d were significantly more frequent in small/middle-sized hospitals compared to large hospitals [13 (61. 9%) vs. 4 (22. 2%), odds ratio = 5.7, 95% confidence interval = 1.4–23.3, *p* = 0.023].

**Conclusions:**

Small/middle-sized hospitals had more frequent interventions within 7 d of broad-spectrum antimicrobial use than large hospitals. More effort to improve AFS is needed among all hospitals.

## Background

Antimicrobial resistance (AMR) is a major threat to global public health, and the World Health Organization published a global action plan to reduce AMR in 2015 [1]. Antimicrobial stewardship (AMS) is an essential part of the control measures for AMR [1]. Several studies have pointed out the importance of implementing AMS in small/middle sized hospitals [2, 3] because small/middle-sized hospitals are the majority of hospital types in many countries, including Japan [4, 5].

The Japan Nosocomial Infections Surveillance (JANIS) reported that the proportion of detected AMR in small/middle-sized hospitals was the same or higher than that in large hospitals [6]. To encourage small/middle-sized hospitals to adopt AMS, the Infectious Diseases Society of America (IDSA) and the Society for Healthcare Epidemiology of America (SHEA) published guidelines for the implementation of AMS [7]. Since 2018, there have been financial incentives for antimicrobial stewardship programs (ASP) under the national health insurance system towards small/middle-sized hospitals as well as large hospitals in Japan. If a hospital sets up an antimicrobial steward team (AST) composed of medical doctors, nurses, pharmacists, and clinical microbiologists, the hospital receives 1000 JPY in revenue per patient [8]. In addition, the importance of antifungal stewardship (AFS) in hospitals is emphasized in some studies because while fungal infections in inpatients are less common than bacterial infections, they are more lethal and costlier to treat [9–11]. The importance of AFS for both patient benefit and cost-saving was well reported in a previous study [12, 13]. The global emergence of antifungal resistance among *Candida* spp. and *Aspergillus* spp. is a growing threat to public health driven largely by the expanding use of antifungals in both clinical and agricultural settings [14].

In Japan, Maeda et al. reported a cross-sectional study on the current situation of AMS [15, 16]. They reported that available human resources, such as medical doctors and pharmacists were important for the implementation of ASP. However, their report did not reveal the actual situation of AMS for individual antibacterial drugs, nor the current situation of AFS in Japan. Moreover, there are few studies evaluating the actual practice of AFS worldwide [17]. To understand the actual situation of AMS and AFS in inpatient settings in both large hospitals and small/middle-sized hospitals in Japan for tackling AMR, we conducted the nationwide cross-sectional study of AMS and AFS in inpatient settings in Japan.

## Methods

### Ethics

The study protocol was approved by the Institutional Review Board at the National Center for Global Health and Medicine (NCGM-G-002473-00) and carried out according to the principles outlined in the Declaration of Helsinki. All participants provided informed consent for participate in our study.

### Study design and population

We conducted a web-based, self-administered, nationwide survey of AMS and AFS in inpatient settings in Japan, targeting hospitals that participated in hospital epidemiology workshops done during July 2018. Survey responses were collected until September 2018. Hospital epidemiology workshops have been held every year in Japan since 2013 for health care workers, such as medical doctors, nurses, pharmacists, and clinical microbiologists, who are involved in infection prevention and control in their respective hospitals. These workshops are organized by the AMR Clinical Reference Center, which was established to lead the fight against AMR according to the National Action Plan on AMR in Japan and is conducted in cooperation with the Japan National Institute of Infectious Disease.

### Data collection

The web-based self-administered questionnaire was developed based on the IDSA and SHEA Guidelines for Developing an Institutional Program to Enhance Antimicrobial Stewardship [7]. The following were collected from the questionnaire: (i) demographic characteristics, including the categorized number of beds (≤ 100 beds, 101 to 200 beds, 201 to 500 beds, ≥501 beds), and the number of staff engaged in ASP, such as medical doctors, including infectious disease specialists (IDS), nurses, including infection control nurses (ICN), pharmacists, including infectious disease chemotherapy pharmacists (IDCP), clinical microbiologists, and nonmedical officer staff; (ii) specific activities regarding AMS, such as prospective audit and feedback (PAF), preauthorization, notification, and interventions within 7 or 28 d for patients on broad-spectrum antimicrobials; and (iii) specific activities regarding AFS, such as PAF, preauthorization, notification, and interventions within 7 or 28 d for patients on antifungals. Notification protocols are of particular importance and mean that although medical doctors remain free to prescribe medications without need for special permissions, they must submit notification forms, which include specific drug-related information, such as duration of use, purpose, and target microorganisms, when prescribing certain antimicrobials and antifungals.

### Definition of variables

All individuals with hospital positions of IDS, ICN, and IDCP were designated under the Japanese Association for Infectious Diseases, the Japanese Nursing Association, and the Japanese Society of Chemotherapy, respectively [18–20]. The broad-spectrum antimicrobials of interest for this study were: 3rd and 4th generation cephalosporins, piperacillin-tazobactam, carbapenem, and intravenous quinolone. These antimicrobials are thought to be important to manage AMR because the abuse of 3rd third-generation cephalosporins is thought to be related to the emergence of extended-spectrum β-lactamase (ESBL)-producing pathogens [21]. Meanwhile, 4th generation cephalosporins, piperacillin-tazobactam, carbapenem, and intravenous quinolone are commonly used for drug-resistant gram-negative bacilli. Antifungals were defined as followed intravenous antifungals: azoles, echinocandins, poriens, and fluoropyrimidines.

Large hospitals and small/middle-sized hospitals were defined as 501 or more beds and 500 beds or less, respectively, based on a previous study [15].

### Statistical analysis

Hospital protocols regarding the use of broad-spectrum antimicrobials and antifungals, including PAF, preauthorization, notification, intervention within 7 d, and intervention within 28 d were compared between large and small/middle-sized hospitals. All analyses were performed using SPSS software version 25 (IBM-SPSS, Inc., Armonk, NY, USA). Bivariate analyses were performed using Fisher’s exact test and the χ^2^ test (categorical variables) with odds ratios (ORs) and 95% confidence intervals (CIs) or Mann–Whitney U test (continuous variables). Two-sided *p* values < 0.05 were considered statistically significant.

## Results

### Characteristics of respondent hospitals

Of the 240 hospitals surveyed, 39 (16.3%) responded. Eighteen (46.2%) were large hospitals, and 21 (53.8%) were small/middle-sized hospitals. In all respondents, the number of hospitals which had a dedicated AST, infection control team, and department of infectious disease were 27 (67.5%), 23 (57.5%), and 11 (27.5%), respectively. Overall, the median (interquartile range [IQR]) number for each staff subtype engaged in ASP were as follows: medical doctors 2 (1–3), IDS 1 (0–1), nurses 1 (1, 2), ICN 1 (1), pharmacists 2 (1, 2), IDCP 0 (0–1), clinical microbiologists 1 (1, 2), and officers 0 (0–1). Twenty-nine (74.4%) hospitals had in-house microbiology laboratories, and 10 (25.6%) hospitals partially utilized out-sourced microbiology laboratories. The median number (IQR) of IDS in small/middle-sized hospitals (0 [0–1]) was statistically lower than that in large hospitals (1 [0.8–2]) (*p* value = 0.035) (Table 1).

**Table 1:** Comparison of characteristics of ASP between small/middle-sized hospitals and large hospitals

	Total *n* = 39		Small/middle hospitals (≤ 500 beds) *n* = 21		Large hospitals (≥ 501 beds) *n* = 18		OR	95% CI	*p* value
Number of beds									
100 ≤ beds	0	(0.0)	0	(0.0)	0	(0.0)			
101 to 200 beds	3	(7.7)	3	(14.3)	0	(0.0)			
201 to 500 beds	18	(46.2)	18	(85.7)	0	(0.0)			
≧ 501 beds	18	(46.2)	0	(0.0)	18	(100.0)			
Department									
AST	27	(67.5)	15	(71.4)	12	(66.7)	1.25	0.32–4.88	1.00
ICT	23	(57.5)	13	(61.9)	10	(55.6)	1.3	0.36–4.68	0.75
Department of infectious disease	11	(27.5)	2	(9.5)	9	(50.0)	0.11	0.019–0.59	0.011
Microbiology laboratory									
In house	29	(74.4)	13	(61.9)	16	(88.9)	0.2	0.037–1.13	0.07
Partially in house	10	(25.6)	8	(38.1)	2	(11.1)	4.92	0.88–27.32	0.07
Number of staffs in AST									
Medical doctors	2	(1–3)	2	(1–3)	2	(1–5)			0.39
Infectious disease specialists	1	(0–1)	0	(0–1)	1	(0.8–2)			0.035
Nurses	1	(1, 2)	1	(1, 2)	1	(1, 2)			0.55
Infection control nurses	1	(1)	1	(1)	1	(1, 2)			0.25
Pharmacists	2	(1, 2)	2	(1–3)	2	(1, 2)			0.77
Infectious disease chemotherapy pharmacists	0	(0–1)	0	(0–1)	0.5	(0–1)			0.53
Clinical microbiologists	1	(1, 2)	1	(1, 2)	1	(1, 2)			0.69
Officers	0	(0–1)	0	(0–1)	0	(0–1)			0.81

Unless otherwise stated, data are presented as n (%)

Continuous variable data are presented as median (IQR)

*ASP* antimicrobial stewardship program, *AST* Antimicrobial stewardship, *ICT* infection control team, *IQR* interquartile range, *OR* odds ratio, *CI* confidential interval

### Intervention for AMS and AFS

Among hospital respondents, a PAF protocol was observed in 23 (59.0%) and 5 (12.8%) hospitals when using broad-spectrum antimicrobials and antifungals, respectively. Preauthorization procedures were observed in 4 (10.3%) and 1 (2.6%) hospital for using broad-spectrum antimicrobials and antifungals, respectively. Notification protocols were present in 37 (94.9%) and 2 (5.1%) hospitals for use of broad-spectrum antimicrobials and antifungals, respectively. The number of hospitals that had an intervention protocol when using broad-spectrum antimicrobials within 7 d was 17 (43.6%) and 34 (87.2%) within 28 d. In contrast, the number of hospitals that had an intervention protocol when using antifungals was 3 (7.7%) within 7 d and 10 (25.6%) within 28 d (Table 2).

**Table 2:** Intervention for AMS and AFS among overall hospitals (*n* = 39)

	Broad-spectrum antibiotics^a^		Antifungals	
PAF				
Yes	23	(59.0)	5	(12.8)
No	16	(41.0)	34	(87.2)
Preauthorization				
Yes	4	(10.3)	1	(2.6)
No	35	(89.7)	38	(97.4)
Notification				
Yes	37	(94.9)	2	(5.1)
No	2	(5.1)	37	(94.9)
Intervention within 7 days				
Yes	17	(43.6)	3	(7.7)
No	22	(56.4)	36	(92.3)
Intervention within 28 days				
Yes	34	(87.2)	10	(25.6)
No	5	(12.8)	29	(74.4)

Unless otherwise stated, data are presented as n (%)

*PAF* prospective audit and feedback, *AMS* antimicrobial stewardship, *AFS* antifungal stewardship

^a^3rd and 4th generation cephalosporins and piperacillin-tazobactam, carbapenem, intravenous quinolone

### Interventions regarding broad-spectrum antimicrobials

The number of hospitals with preauthorization and notification protocols, respectively, regarding the investigated antibiotics were as follows: broad-spectrum antimicrobials overall 4 (10.3%) and 37 (94.9%); carbapenem 2 (5.1%) and 34 (87.2%); 3rd generation cephalosporin 0 (0%) and 0 (0%); 4th generation cephalosporin 0 (0%) and 10 (25.6%); piperacillin/tazobactam 0 (0%) and 17 (43.6%); and intravenous quinolone 3 (7.7%), and 18 (46.2%). Regarding preauthorization and notification protocols, there were no significant differences between small/middle-sized hospitals and large hospitals. The numbers for hospitals that had intervention procedures within 7 d and 28 d, respectively, for each investigated antibiotic were as follows: broad-spectrum antimicrobials overall 17 (43.6%) and 34 (87.2%); carbapenem 16 (41.0%) and 34 (87.2%); 3rd generation cephalosporin 1 (2.6%) and 11 (28.2%); 4th generation cephalosporin 7 (17.9%) and 20 (51.3%); piperacillin/tazobactam 12 (30.8%) and 23 (59.0%); and intravenous quinolone 13 (30.8%) and 22 (56.4%). Intervention procedures to use broad-spectrum antimicrobials within 7 d were statistically more frequent in small/middle-sized hospitals than in large hospitals with findings as follows: overall, OR = 5.7, 95% CI = 1.4–23.5, *p* = 0.023; carbapenem, OR = 4.7, 95% CI = 1.1–19.1, *p* = 0.049; piperacillin/tazobactam, OR = 7.3, 95% CI = 1.3–39.9, *p* = 0.018; and intravenous quinolone, OR = 8.8, 95% CI = 1.6–48.2, *p* = 0.008. There was no significant difference between small/middle-sized hospitals and large hospitals regarding intervention procedures to use broad-spectrum antimicrobials within 28 d (Table 3).

**Table 3:** Comparison of intervention for AMS between small/middle-sized hospitals and large hospitals

	Total *n* = 39		Small/middle-sized hospitals (≤ 500 beds) *n* = 21		Large hospitals (≥ 501 beds) *n* = 18		OR	95% CI	*p* value
Intervention within 7 days									
Overall	17	(43.6)	13	(61.9)	4	(22.2)	5.7	1.4–23.5	0.023
Carbapenem	16	(41.0)	12	(57.1)	4	(22.2)	4.7	1.1–19.1	0.049
3rd generation cephalosporine	1	(2.6)	1	(4.8)	0	(0)			
4th generation cephalosporine	7	(17.9)	6	(28.6)	1	(5.6)	6.8	0.7–63.1	0.098
Piperacillin/tazobactam	12	(30.8)	10	(47.6)	2	(11.1)	7.3	1.3–39.9	0.018
Intravenous quinolone	13	(30.8)	11	(52.4)	2	(11.1)	8.8	1.6–48.2	0.008
Intervention within 28 days									
Overall	34	(87.2)	20	(95.2)	14	(77.8)	5.7	0.6–56.7	0.16
Carbapenem	34	(87.2)	20	(95.2)	14	(77.8)	5.7	0.6–56.7	0.16
3rd generation cephalosporine	11	(28.2)	7	(33.3)	4	(22.2)	1.8	0.4–7.4	0.50
4th generation cephalosporine	20	(51.3)	13	(61.9)	7	(38.9)	2.6	0.7–9.3	0.21
Piperacillin/tazobactam	23	(59.0)	14	(66.7)	9	(50.0)	2.0	0.6–7.3	0.34
Intravenous quinolone	22	(56.4)	14	(66.7)	8	(44.4)	2.5	0.7–9.2	0.21

Unless otherwise stated, data are presented as n (%)

*AMS* antimicrobial stewardship, *OR* odds ratio, *CI* confidential interval

### Intervention protocols for antifungals

The following is the number of small/middle hospitals with the following protocols for antifungals: PAF (3; 14.3%), preauthorization (1; 4.8%), notification (0; 0%), intervention within 7 d (3; 14.3%), and intervention within 28 d (7; 33.3%). For large hospitals, findings were as follows: PAF (2; 11.1%), preauthorization (0; 0%), notification (2; 11.1%), intervention within 7 d (0; 0%), and intervention within 28 d (3; 16.7%). There was no significant difference between small/middle-sized hospitals and large hospitals regarding intervention protocols for antifungals (Table 4).

**Table 4:** Comparison of intervention for AFS between small/middle-sized hospitals and large hospitals

	Total *n* = 39		Small/middle-sized hospitals (≤ 500 beds) *n* = 21		Large hospitals (≥ 501 beds) *n* = 18		OR	95% CI	*p* value
Intervention within 7 days									
Overall	3	(7.7)	3	(14.3)	0	(0)			
Azole	2	(5.1)	2	(9.5)	0	(0)			
Echinocandin	2	(5.1)	2	(9.5)	0	(0)			
Porien	2	(5.1)	2	(9.5)	0	(0)			
Fluoropyrimidine	1	(2.6)	1	(4.8)	0	(0)			
Intervention within 28 days									
Overall	10	(25.6)	7	(33.3)	3	(16.7)	2.5	(0.5–11.6)	0.29
Azole	9	(23.1)	6	(28.5)	3	(16.7)	2.0	(0.4–9.5)	0.46
Echinocandin	9	(23.1)	6	(28.5)	3	(16.7)	2.0	(0.4–9.5)	0.46
Porien	8	(20.5)	5	(23.8)	3	(16.7)	1.6	(0.3–7.7)	0.70
Fluoropyrimidine	4	(10.3)	2	(9.5)	2	(11.1)	0.8	(0.1–6.7)	1.00

Unless otherwise stated, data are presented as n (%)

*AMS* antimicrobial stewardship, *OR* odds ratio, *CI* confidential interval

## Discussion

Our findings revealed that the small/middle-sized hospitals had more frequent interventions regarding broad-spectrum antimicrobial use within 7 days in inpatient settings. Regarding interventions within 7 days when using carbapenem, piperacillin/tazobactam, and intravenous quinolone, small/middle-sized hospitals were more involved. In a previous Japanese study, large hospitals tended to have more ASP and the reason was thought to be a greater number of full-time equivalent pharmacists compared to small/middle-sized hospitals [16]. We think that one of the reasons for the inconsistency between the results of our study and those of the previous study is participants. Although the participants of previous study were certified members of the Japanese College of Infection Control Doctors in nationwide, those in our study were highly motivated medical staffs who participated in hospital epidemiology workshops during July 2018. The reason small/middle-sized hospitals intervened more frequently in our study is that small hospitals may be more likely to change their practices if they have highly motivated personnel. Another study reported that a barrier to ASP implementation in small hospitals was ﻿a lack of dedicated IDS to support the ASP [3]. In our study, small/middle-sized hospitals tended to have fewer IDS, but had more frequent interventions compared to large hospitals. In small/middle-sized hospitals, it may be that pharmacists play a key role in AST, especially regarding interventions for antimicrobial use, despite the lack of IDS. We hypothesize that in small/middle-sized hospitals, it is difficult for an IDS to dedicate an adequate amount of time for AST activities because of their many tasks in Japan. However, pharmacists have more time to participate in AST and thus, are more often designated for AST activities compared to their IDS counterparts. This situation may suggest that pharmacists have more initiative for AST activities in small/middle-sized hospitals than in large hospitals. This may lead to prompt actions for intervention in cases of inappropriate antimicrobial use. In fact, it has been reported that pharmacists play a central role in AST [22]. On the other hand, the number of hospitals that had intervention protocols within 7 days of using of 3rd and 4th generation cephalosporin was small in both small/middle-sized hospitals (1/21 [4.8%], 6/21 [28.6%]) and large hospitals (0/18 [0%], 1/18 [5.6%]). AMS for 3rd generation cephalosporins was thought to be related to the decrease of ESBL-producing pathogens [21]. Because JANIS reported that the proportion of *Escherichia coli* resistance to cefotaxime increased from 23.3 to 27.5% between the years 2014–2018 in Japan [23], prompt intervention protocols for not only 4th generation cephalosporins, but also 3rd generation cephalosporins should be emphasized.

Our study showed that most hospitals, regardless of size, appeared to provide few interventions regarding antifungal use in inpatient settings (intervention within 7 d: 3 [14.3%], 0 [0%], intervention within 28 d: 7 [33.3%], 3 [16.7%]). The reason for this is thought to be due to a lack of human resource power and the low incidence of fungal infections compared to bacterial infections in inpatient settings [23]. However, the importance of AFS for both patient benefit and cost-saving was well reported in a previous study [12, 13]. Based on the national database of health insurance claims and specific health checkups in Japan, although the cost of antimicrobials decreased from 163 billion dollars in 2013 to 121 billion dollars in 2017, the cost of antifungals increased from 34 billion dollars in 2013 to 50 billion dollars in the same year [24]. A previous observational study in Japan pointed out that AFS was important to reduce the cost of antifungal use [25]. In terms of not only patient benefit, but also the cost savings, we think that the importance of appropriate use of antifungal agents is paramount. As a way to facilitate AFS, in previous study, it was effective for decrease antifungals use making guideline for antifungal infection and PAF with bed-side visiting by IDSs [10]. However, this may be difficult in hospitals with few or no IDSs. Because of this, we suggest pharmacist-centered monitoring and active PAF based on the result of previous our study [26]. Although this was a study on the appropriate use of carbapenems, we believe that a similar method would be effective for the appropriate use of antifungals. In addition, we suggest closer communication with clinical departments which antifungals are more frequently used. In this study, it was found that both AMS and AFS were not sufficiently implemented. One of the reasons for this may be insufficient manpower of AST staff in both large hospitals and small/middle-sized hospitals in Japan, especially in small/middle-sized hospitals. One of the efficient AMS and AFS activities with limited resources, we suggest the education about the principle of appropriate use of antimicrobials and antifungals, to ensure that appropriate use is made without the intervention of AST staff.

There were several limitations to our study. First, this study was based on a self-reported questionnaire, so there may be differences between the results of our study and actual hospital activity regarding AMS and AFS. Second, we have the possibility of respondent bias due to a low proportion and limited number of respondents in that a respondent facility which also participated in the hospital-epidemiology workshop might be a motivated cohort. The results of our study had the possibility of being overestimated, compared to one that directly observed all targeted institutions. Third, our study did not include oral both antimicrobials and antifungals. Both AMS and AFS toward oral antimicrobials and antifungals are also important and requires further investigation including them. Fourth, although the patients’ characteristics and the antimicrobials and antifungals used may be very different in university hospitals, cancer centers, and rehabilitation hospitals, there are no detail of characteristics of the participated the hospitals in this study. To know the actual situation of AMS and AFS, further investigation including the detail of characteristics of the participated the hospitals with larger sample size are thought to be needed.

## Conclusions

This study is the nationwide cross-sectional study to reveal the actual situation of AMS and AFS in both large hospitals and small/middle-sized hospitals in inpatient settings in Japan. Small/middle-sized hospitals tended to have more frequent interventions for broad-spectrum antimicrobial use within 7 days compared to large hospitals. This result might reflect human resource allocation, especially regarding pharmacists. Our study also revealed that there was more effort to improve interventions for the use of antifungals among both small/middle-sized and large hospitals. A further study collecting actual activity of ASP with a large number of hospitals is needed to provide more effective AMS and AFS to tackle AMR in Japan.

## Data Availability

The datasets used and/or analyzed during the current study are available from the corresponding author on reasonable request.

## Abbreviations

AMS: Antimicrobial stewardship
AFS: Antifungal stewardship
AMR: Antimicrobial resistance
ASP: Antimicrobial stewardship programs
AST: Antimicrobial steward team
JANIS: Japan Nosocomial Infections Surveillance
IDSA: Infectious Disease Society of America
SHEA: Society for Healthcare Epidemiology of America
IDS: Infectious disease specialist
ICN: Infection control nurses
PAF: Prospective audit and feedback
IDCP: Infectious disease chemotherapy pharmacists
ESBL: Extended-spectrum β-lactamase
